# Testing umbilical cords for funisitis due to Treponema pallidum infection, Bolivia.

**DOI:** 10.3201/eid0605.000507

**Published:** 2000

**Authors:** J Guarner, K Southwick, P Greer, J Bartlett, A Santander, S Blanco, V Pope, W Levine, S Zaki

**Affiliations:** Centers for Disease Control and Prevention, Atlanta, Georgia 30333, USA. jqg8@cdc.gov

## Abstract

To establish the frequency of necrotizing funisitis in congenital syphilis, we conducted a prospective descriptive study of maternal syphilis in Bolivia by testing 1,559 women at delivery with rapid plasma reagin (RPR). We examined umbilical cords of 66 infants whose mothers had positive RPR and fluorescent treponemal antibody absorption tests. Histologic abnormalities were detected in 28 (42%) umbilical cords (seven [11%] had necrotizing funisitis with spirochetes; three [4%] had marked funisitis without necrosis; and 18 [27%] had mild funisitis), and 38 [58%] were normal. Of 22 umbilical cords of infants from mothers without syphilis (controls), only two (9%) showed mild funisitis; the others were normal. Testing umbilical cords by using immunohistochemistry is a research tool that can establish the frequency of funisitis due to Treponema pallidum infection.


### Testing Umbilical Cords for Funisitis due to *Treponema pallidum* Infection, Bolivia

**Jeannette Guarner,* Karen Southwick,* Patricia Greer,* Jeanine Bartlett,* Martha Fears,* Ana Santander,† Stanley Blanco,† Victoria Pope,* William Levine,* and Sherif Zaki***
*Centers for Disease Control and Prevention,
Atlanta, Georgia, USA; †MotherCare, La Paz, Bolivia

To establish the frequency of necrotizing funisitis in congenital syphilis, we conducted a prospective descriptive study of maternal syphilis in Bolivia by testing 1,559 women at delivery with rapid plasma reagin (RPR). We examined umbilical cords of 66 infants whose mothers had positive RPR and fluorescent treponemal antibody absorption tests. Histologic abnormalities were detected in 28 (42%) umbilical cords (seven [11%] had necrotizing funisitis with spirochetes; three [4%] had marked funisitis without necrosis; and 18 [27%] had mild funisitis), and 38 [58%] were normal. Of 22 umbilical cords of infants from mothers without syphilis (controls), only two (9%) showed mild funisitis; the others were normal. Testing umbilical cords by using immunohistochemistry is a research tool that can establish the frequency of funisitis due to *Treponema pallidum* infection.

### Introduction

Maternal and congenital syphilis are public health problems not only in developing countries, but worldwide. Even in the United States, congenital syphilis continues to occur (1). In Bolivia, the reported rate of congenital syphilis in 1994 was 3.1 per 1,000 live births (2).

Congenital syphilis usually results from transplacental passage of *Treponema pallidum* to the fetus, causing infection of the placenta and umbilical cord. For the fetus, the likelihood of infection is greatest when the mother has primary or secondary syphilis in the last two trimesters of pregnancy (3). At birth, only half of infected newborns have clinical signs of disease (3). Placental infection is not always present, and affected placentas show varying degrees or combinations of three basic histologic findings (4-6): focal proliferative villitis, endovascular and perivascular proliferative changes in villous vessels, and relative immaturity of villi. The umbilical cord may have inflammatory infiltrates around vessels, a condition known as necrotizing funisitis (4,6). To identify which infants without clinical signs have congenital infection, laboratory tests of maternal or newborn sera, as well as of the placenta and umbilical cord, have been developed (7). Several techniques have been used to detect spirochetes in the placenta, including silver stains, immunohistochemistry (IHC), and direct fluorescence antibody testing (DFA) (6,8). The latter two are specific for syphilis because they use antibodies against *T. pallidum*. IHC also allows visualization of morphologic features of the tissue, thus permitting localization of the microorganism in tissue structures. Previous pathologic studies of placentas and umbilical cords have been retrospective and based on small population samples and have presented data from specimens analyzed because of specific perinatal problems (5,6). Thus, the frequency of histopathologic changes in the placenta and umbilical cord has not been defined.

As part of a study to document prevalence of maternal syphilis in Bolivia, we assessed the histopathologic features of umbilical cords in a cohort of live-born and stillborn infants of women with syphilis and a sample of infants born to women without syphilis. We established the frequency of histopathologic abnormalities in umbilical cords from neonates of mothers with

Address for correspondence: Jeannette Guarner, Infectious Disease Pathology Activity, Centers for Disease Control and Prevention, Mailstop G32, 1600 Clifton Rd., N.E., Atlanta, GA 30333; Fax: (404) 639-3043; e-mail: jqg8@cdc.gov.

syphilis, compared these results with those of the sample without syphilis, and analyzed the usefulness of IHC and DFA for detection of *T. pallidum*.

### Materials and Methods

All women delivering in seven hospitals in Bolivia from June to November 1996 were eligible for the study; 1,559 women consented to participate (participation rate 63%). Women were given a risk factor questionnaire, and blood was obtained for a rapid plasma reagin (RPR) test. If a woman had a positive RPR result (1:1 dilution or above), both she and her infant were treated with penicillin according to Centers for Disease Control and Prevention (CDC) treatment guidelines (9). Maternal syphilis was defined as positive RPR and treponemal antibody absorption test results from one of two Bolivian reference laboratories chosen on the basis of their performance on a proficiency panel (CDC, Atlanta, GA). For quality control, sera from all women with syphilis and a systematic sample of 10% of the women without syphilis were tested at CDC. Umbilical cords from the subset of infants whose mothers' sera had been tested at CDC were examined; this subset included 66 umbilical cords from infants born to women with syphilis, as well as 22 control umbilical cords.

Immediately after delivery, a section of umbilical cord (approximately 3 cm to 4 cm long) was collected from the end closest to the infant, fixed in 10% formalin, and transported to CDC, where it was embedded in paraffin. We examined at least two sections from 49 cords and three to five sections from 39 cords. The tissues were studied by using hematoxylin and eosin stain, and DFA and IHC tests were performed for *T. pallidum*. The extent and location of the inflammatory process were evaluated histologically. The cords were classified into two categories according to the intensity of inflammation: mild funisitis if <10 inflammatory cells (polymorphonuclear leukocytes, lymphocytes, or macrophages) were seen at 40x magnification and marked funisitis if $\geq$10 inflammatory cells were seen. Necrosis, which was recorded independent of the degree of inflammation, was defined as presence of eosinophilic cellular debris surrounded by inflammation.

IHC was performed as described (10): 3-µm sections of cord were deparaffinized, rehydrated, placed in a DAKO autostainer (DAKO Corporation, Carpinteria, CA), digested in 0.1 mg/mL Proteinase K (Boehringer-Mannheim Corporation, Indianapolis, IN), and later blocked with normal swine serum. After a 1-hr incubation with polyclonal rabbit antibody against *T. pallidum* (CDC, Atlanta, GA), detection was done with a biotinylated anti-rabbit antibody, strepavidin-alkaline phosphatase complex, and naphthol/fast red substrate (DAKO Corporation). Sections were counterstained in Meyer's hematoxylin (Fisher Scientific, Pittsburgh, PA). Positive controls were tissue sections of a testis from a *T. pallidum*-infected rabbit and of liver and gastrointestinal tract from a human case of congenital syphilis. Negative controls consisted of each patient's tissue sections incubated with normal rabbit serum and an unrelated polyclonal antibody instead of the polyclonal antibody against *T. pallidum*. Positive staining by IHC was considered only if intact spirochetes were identified. In four cases, more sections from the umbilical cord were submitted for study by IHC and DFA because fine and coarse red granular staining was considered questionable for infection.

DFA was performed as described (6): 3-µm sections were deparaffinized, rehydrated through graded alcohol passages, and then digested with trypsin. Next, slides were stained with a fluorescein-labeled monoclonal conjugate to *T. pallidum* (CDC, Atlanta, GA). Slides were mounted and read with an epi-illuminated fluorescent microscope. The same positive control was used for both IHC and DFA. Positive results were reported if intact spirochetes were identified. Additional sections of five cords were submitted for DFA and IHC study because fluorescent staining was considered questionable for infection.

Frequencies of the histologic measurements were analyzed. Univariate analysis was done with the chi-square statistic or Fisher's exact test (if expected cell values were <5) by Epi-Info (CDC, Atlanta, GA) and SAS (Cary, NC) statistical packages.

### Results

The histologic examination of umbilical cords from infants born to mothers with syphilis demonstrated the following: seven cords (11%) with marked funisitis, necrosis (necrotizing funisitis) and treponemes (five by IHC and DFA; two by IHC only); marked funisitis without necrosis or spirochetes in three (4%) cases; mild funisitis in 18 (27%) cases; and no histopathologic abnormality in 38 (58%) cases. Histopathologic studies of umbilical cords from infants of mothers without syphilis identified two (9%) cases with mild funisitis; the other 20 (91%) had normal histologic features. The association of treponemes, acute and chronic inflammation, and necrosis was different in the umbilical cord structures (Table 1).

Marked funisitis was associated with both maternal syphilis and presence of treponemes in the cord. All 10 umbilical cords with marked funisitis were from infants whose mothers had syphilis; these cords were more likely to have *T. pallidum* identified by IHC or DFA than were those that did not have marked funisitis (70% vs. 4%; odds ratio [OR] 41.2; 95% confidence interval [CI] 5.5-405.7). In six of the cases of marked funisitis, acute and chronic inflammatory infiltrates were observed in one or more vessels and in the Wharton's jelly (Figure 1); all these cords were positive for *T. pallidum* by either IHC or DFA. Marked funisitis was also diagnosed in one case with acute and chronic inflammation in a vein but not in the Wharton's jelly; this cord showed spirochetes by IHC. In the seven cases described, inflammation was accompanied by necrosis. Three cords with marked funisitis had acute inflammation in a vessel but no necrosis or inflammation in the Wharton's jelly. All three were negative for *T. pallidum* by IHC and DFA.

Mild funisitis was diagnosed in 20 (30%) cords; 18 were from infants whose mothers had syphilis; none contained spirochetes. Two cords from infants born to control mothers had mild funisitis. A higher percentage of infants with mild funisitis than infants with normal umbilical cords had mothers with syphilis (90% vs. 66%; OR 4.7; p=0.04). Of cords from infants born to RPR-positive mothers, 38 (58%) had normal histologic features; one of these contained spirochetes identified by DFA. However, umbilical cords with mild funisitis did not differ substantially from normal cords with respect to presence of treponemes (5% vs. 3%).

Necrosis was identified in seven cases with marked funisitis and one case with mild funisitis. All eight cases with necrosis of the umbilical cord were from infants of mothers with syphilis; none of the controls had necrosis. Necrosis in any of the umbilical vessels or amnion was strongly associated with the presence of *T. pallidum* by IHC or DFA (seven of eight cases). When necrosis

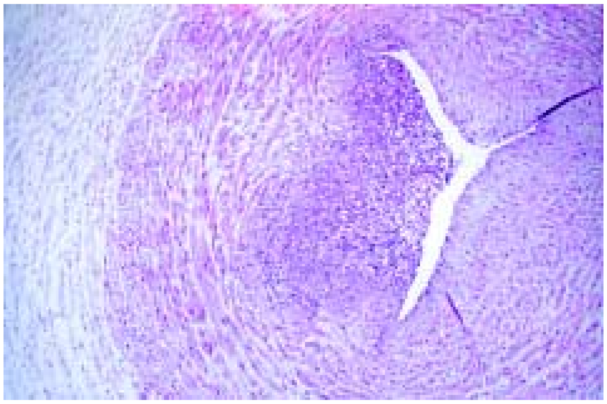

Figure 1. Umbilical artery, showing marked inflammation in the intima of the vessels.

Table 1. Inflammation, necrosis, and treponemes in umbilical cords from infants with congenital syphilis, Bolivia

| Umbilical cord structure | Histologic parameter | Specimens with treponemes No. pos./no. tested (%) (N = 9) | Specimens without treponemes No. pos./no. tested (%) (N = 79) | Odds ratio (95% confidence intervals) |
|---|---|---|---|---|
| Umbilical artery[a] | Acute inflammation | 3/8 (38) | 6/78 (8) | 7.2 (1.0-49) |
| | Chronic inflammation | 3/8 (38) | 4/78 (5) | 11.1 (1.5-89) |
| | Necrosis | 2/8 (25) | 0/78 (0) | undefined[b] |
| | Normal | 3/8 (38) | 65/78 (83) | 0.1 (0.0-0.4) |
| Umbilical vein | Acute inflammation | 7/9 (78) | 15/78 (19) | 14.7 (2.4-115) |
| | Chronic inflammation | 5/9 (56) | 5/78 (6) | 18.3 (3.0-125) |
| | Necrosis | 5/9 (56) | 0/78 (0) | undefined[c] |
| | Normal | 1/9 (11) | 50/78 (64) | 0.1 (0.0-0.6) |
| Wharton's jelly | Acute inflammation | 8/9 (89) | 12/79 (15) | 44.7 (4.8-1,043) |
| | Chronic inflammation | 5/9 (56) | 4/79 (5) | 23.4 (3.6-178) |
| | Normal | 1/9 (11) | 6/79 (8) | 0.03 (0.0-0.3) |

[a]The umbilical artery was missing in two specimens, and the umbilical vein was missing in another.
[b]p=0.01.
[c]$p<0.001$.

was present in one structure, it was usually present in others; for example, necrosis of the umbilical vein was present in five (71%) of the seven cords with amnion necrosis, but in none of the 78 cords without amnion necrosis (OR undefined; $p<0.001$). Twelve umbilical cords showed neovascularization. Eight of these cords were from infants of mothers with syphilis (12%) and four were from controls (18%). Five cords had neovascularization accompanied by inflammation, and spirochetes were identified in one. Neovascularization was not associated with funisitis.

IHC demonstrated intact spirochetes and varying degrees of granular staining in seven (10.6%) umbilical cords of infants whose mothers had syphilis. In four cases, the *T. pallidum* level was high ($\geq$10 bacteria per field at 40x magnification and presence in $\geq$2 of the sections examined). In three cases, the spirochete level was lower, with <9 bacteria per field at 40x magnification and presence in only one section. Granular staining indicative of infection was present in nine cases: four by IHC and five by DFA. For all these cases, extra tissue was submitted, and in only one were spirochetes identified on reexamination.

With IHC, we were able to define the location of intact spirochetes and granular staining in the umbilical cord. In the seven positive cords, intact spirochetes were seen where there was less inflammatory infiltration (Figure 2). Granular staining was seen in the areas with marked inflammation, sometimes accompanied by intact bacteria. In the necrotic areas, no spirochetes were demonstrated, and little or no granular staining was seen (Figure 3).

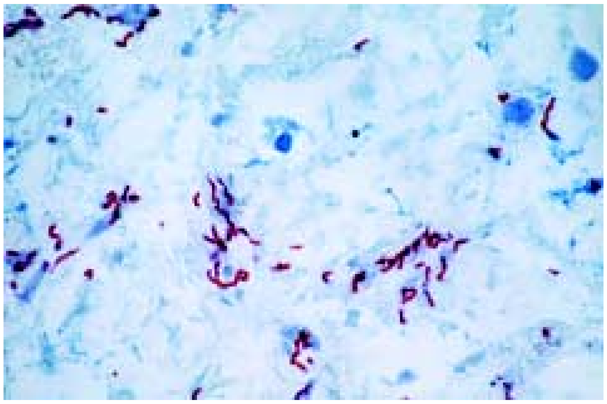

Figure 2. Immunohistochemistry for *Treponema pallidum* in an area with few inflammatory infiltrates and multiple spirochetes.

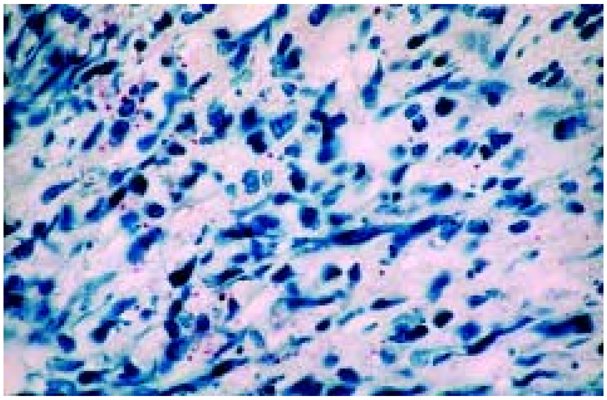

Figure 3. Immunohistochemistry for *Treponema pallidum* in an area with marked inflammatory infiltrate with granular staining; no intact spirochetes are noted.

Comparison of IHC and DFA results showed that all seven umbilical cords positive by IHC had marked funisitis and were from infants of mothers with syphilis; five of these were also positive by DFA. Two cords were positive by DFA but negative by IHC. One was from an infant whose mother had syphilis, and the other was from a control. Both these cords had normal histopathologic features.

Information about antibiotic treatment for syphilis or other conditions near term or during labor and delivery (including premature rupture of membranes) was incompletely recorded in the medical charts. Penicillin treatment for syphilis during pregnancy was documented for two women: one 6 months and the other 6 weeks before delivery. Neither umbilical cord had funisitis. Birth weight, gestational age, and physical findings for infants born to mothers with syphilis have been described (11). Of the 66 women with syphilis, 6 (9%) had stillborn infants, 1 had marked funisitis with spirochetes demonstrated by both DFA and IHC, 1 had mild funisitis with a positive DFA, and 4 had normal umbilical cord histopathologic features with no spirochetes noted on IHC or DFA.

### Conclusions

We found histologic abnormalities ranging from mild to marked funisitis in 42% of umbilical cords from infants of mothers with syphilis. The presence of *T. pallidum* in umbilical cords was strongly associated with marked inflammation and necrosis (necrotizing funisitis). Newborns whose cords showed marked funisitis, necrosis, and spirochetes are assumed to have congenital syphilis.

Necrotizing funisitis, which was described at the beginning of the 20th century, has been linked to syphilitic infection and high fetal-infant death rates (4,6). The disorder refers to a deep-seated inflammatory process in the matrix of the umbilical cord, which may be accompanied by phlebitis and thrombosis. Necrotizing funisitis is thought to result from diffusion of amniotic fluid leukotoxin, which destroys the fetal neutrophils migrating toward the amniotic cavity (12). The presence of spirochetes in umbilical cords with necrotizing funisitis has been reported in 40% (4,13) to 90% of cases (6).

We found three cords with marked funisitis but no necrosis. In these three cases, no spirochetes were present, although the cords came from infants of mothers with syphilis. We also found 18 umbilical cords with mild funisitis, all from infants born to women with syphilis. In these cords we attributed inflammation, whether mild or marked, to *T. pallidum* infection. Some authors distinguish necrotizing funisitis from acute funisitis, since the latter is characterized by acute inflammation without necrosis and can be associated with infections other than syphilis (6). We found two umbilical cords with mild funisitis from infants of women without syphilis; the inflammation in these cases could have been secondary to other pathologic conditions, such as premature rupture of membranes or bacterial infection other than syphilis (4,12).

*T. pallidum* infection is difficult to confirm in infants whose cords do not show spirochetes, whether they have marked funisitis without necrosis, mild funisitis, or necrosis with minimal inflammation. We noted pathologic changes in 42% of umbilical cords from infants born to women with syphilis. These infants may have been infected even though no spirochetes were identified. These data support the CDC recommendation to provide immediate penicillin treatment at delivery for all newborn infants born to women with untreated or inadequately treated syphilis during pregnancy (9).

In an ideal study of congenital syphilis, umbilical cords would be examined in conjunction with the placenta (5), and fetal outcome would be taken into account (13). However, good sensitivity for detecting *T. pallidum* from umbilical cords only has been reported (6). In epidemiologic field studies such as ours, a small portion of umbilical cord is easier to obtain and transport than the entire placenta and permits histopathologic and immunohistochemical analysis of tissues, which can be helpful in assessing populations at risk for congenital syphilis.

We compared our results of umbilical cord abnormalities from infants whose mothers had syphilis with those published by other authors (Table 2) (5,6). Qureshi et al. described placental pathology data from 25 of 162 cases of syphilis in which placentas were selectively sent to the pathology laboratory by the obstetricians (5). Schwartz et al. limited their selection to 25 women with active syphilis (6). The frequency of histologic changes in our cohort is similar to the findings of Qureshi et al. (5). Schwartz et al. (6) reported a lower percentage of normal cords and higher frequency of necrotizing funisitis and spirochetes. The other authors studied a small, selected group of umbilical cords from patients with known early syphilis and high RPR titers; in contrast, our study included all women with reactive RPR tests and with both early and late syphilis. In the latter group, transmission to the fetus is less likely (7).

Up to 75% of pregnant women with active syphilis have stillbirths (6). In the group of patients we studied, six women had documented stillbirths; of the six stillborn infants, two had histologic abnormalities of the umbilical cord and *T. pallidum* was demonstrated, so syphilis was assumed to be the cause of fetal death. In the other four cases, death cannot be attributed entirely to syphilis, especially in communities where prenatal care is poor. We found no correlation between outcome of pregnancy and umbilical cord abnormalities; however, our study may be limited by the small number of stillborn infants.

Table 2. Comparison of three studies of umbilical cord abnormalities of infants born to women with syphilis

| Pathologic features | Schwartz (6) | Qureshi (5)[a] | Guarner et al. |
|---|---|---|---|
| No. of cords | 25 (%) | 25 (%) | 66 (%) |
| Normal histology | 12 (48) | 14 (56) | 38 (58) |
| Funisitis | 13 (52) | 11 (44) | 28 (42) |
| Inflammation only | 4 (16) | 8 (32) | 20 (30) |
| Necrosis | 9 (36) | 3 (12) | 8 (12) |
| Spirochetes identified | 22 (88) | --- | 7 (11) |
| Stillbirths | 18 (72) | 4 (16) | 6 (9) |

[a]Data extracted from text.

Most previous studies of tissues have used the silver stain (Steiner) (4,6) or DFA (6) to detect spirochetes. Problems encountered when using the silver stains include lack of specificity—most bacteria stain with Steiner—and silver precipitation frequently causing high background, which decreases sensitivity. The use of specific antibodies to *T. pallidum* increases the specificity, while sensitivity is heightened by several detection methods. DFA, which has been compared with Steiner stains for the study of syphilis in umbilical cords, has slightly better sensitivity and specificity (6). IHC had advantages for the diagnosis of congenital syphilis. We were able to define the exact location of the spirochetes in the umbilical cord; intact spirochetes were found in areas of less inflammation, but *T. pallidum* was not seen in necrotic areas. We also described granular staining, which probably corresponded to either spirochetes cut in different planes or antigenic fragments of *T. pallidum*. Other methods, such as polymerase chain reaction (PCR), can be used to enhance sensitivity (8); however, with PCR and DFA, tissue cannot be observed histologically. Therefore, we found that IHC correlated better with marked funisitis.

Although marked funisitis with necrosis (necrotizing funisitis) was identified in only 12% of umbilical cords from infants of mothers with syphilis, it is highly associated with the presence of spirochetes by either IHC or DFA. Abnormal histopathologic results are found in umbilical cords from infants of mothers with syphilis; however, normal results are found in more than half the cases. Our study demonstrates the value of histologic and IHC analysis of umbilical cords for epidemiologic research. However, routine clinical management of patients, especially in resource-poor settings, should not depend on examination of umbilical cords. Congenital syphilis can be controlled and prevented by widespread RPR testing and penicillin treatment during prenatal care.

This project was approved by the Bolivian Ministry of Health and Social Welfare, participating local institutions, the Office for Protection from Research Risks of the National Institutes of Health, and the Institutional Review Board of the Centers for Disease Control and Prevention. This study was made possible through support provided by John Snow, Inc., MotherCare Project and the Office of Health and Nutrition Bureau for Global Programs, Field Support and Research, US Agency for International Development, under the terms of contract number HRN-Q-00-3039-00.

Dr. Guarner is a staff pathologist in the Division of Viral and Rickettsial Diseases, National Center for Infectious Diseases, Centers for Disease Control and Prevention. Her research interests include the acute and chronic effects of infectious agents, especially bacteria and parasitic infections.

### References

1. Congenital syphilis—United States, 1998. MMWR Morb Mortal Wkly Rep 1999;48:757-61.
2. Secretaria Nacional de Salud. Plan a mediano plazo (PMPII) para la vigilancia y prevención del VIH/SIDA: Bolivia 1995-1997. Control de Enfermedades y Riesgo, Programa Nacional de ETS-SIDA. La Paz, Bolivia: the Secretariat; 1995.
3. Zenker PN, Berman SM. Congenital syphilis: trends and recommendations for evaluation and management. Pediatr Infect Dis J 1991;10:516-22.
4. Fojaco RM, Hensley GT, Moskowitz L. Congenital syphilis and necrotizing funisitis. JAMA 1989;261:1788-90.
5. Qureshi F, Jacques SM, Reyes MP. Placental histopathology in syphilis. Hum Pathol 1993;24:779-84.
6. Schwartz DA, Larsen SA, Beck-Sague C, Fears M, Rice RJ. Pathology of the umbilical cord in congenital syphilis: analysis of 25 specimens using histochemistry and immunofluorescent antibody to *Treponema pallidum*. Mod Pathol 1995;26:784-91.
7. Evans HE, Frenkel LD. Congenital syphilis. Clin Perinatol 1994;21:149-62.
8. Genest DR, Choi-Hong SR, Tate JE, Qureshi F, Jacques SM, Crum C. Diagnosis of congenital syphilis from placental examination: comparison of histopathology, Steiner stain and polymerase chain reaction for *Treponema pallidum* DNA. Hum Pathol 1996;27:366-72.
9. Centers for Disease Control and Prevention. 1998 Sexually transmitted diseases treatment guidelines. MMWR Morb Mortal Wkly Rep 1998;47:No. RR-1.
10. Guarner J, Greer PW, Bartlett J, Ferebee T, Fears M, Pope V, Zaki SR. Congenital syphilis in a newborn: an immunopathologic study. Modern Pathol 1999;12:82-7.
11. Southwick KL, Blanco S, Santander A, Estenssoro M, Torrico F, Seoane G, et al. Maternal and congenital syphilis in Bolivian infants: seroprevalence and risk factors. WHO Bulletin. In press 2000.
12. Hyde SR, Altshuler G. Placentitis. In: Connor DH, Chandler FW, Schwartz FW, Manz HJ, Lack EE, editors. Pathology of infectious diseases. Hong Kong: Appleton & Lange; 1997. p. 1675-93.
13. Young SA, Crocker DA. Occult congenital syphilis in macerated stillborn fetuses. Arch Pathol Lab Med 1994;118: 44-7.
